# Molecular docking analysis of breast cancer target RAC1B with ligands

**DOI:** 10.6026/9732063002001467

**Published:** 2024-11-05

**Authors:** Kajal Verma, Lakshmi Pillai

**Affiliations:** 1 Institute of Sciences, SAGE University, Indore Madhya Pradesh, India 452020

**Keywords:** Structure based drug design, breast cancer, RAC1B, molecular docking, ADMET, molecular dynamics simulation

## Abstract

Breast cancer is a malignant neoplasm that arises from the breast tissue, and the best chemotherapy preventive approach is to identify potent inhibitors. In this study, focusing on the Rac1b protein may be an effective approach to developing drug alternatives to treat breast cancer, and we have employed structure-based drug design with the available drugs. Afterwards, molecular docking was used to identify novel inhibitors, and in order to compute the drug likeness and medicinal chemistry, the best-docked complex was put through ADMET studies followed by molecular dynamics simulations to check the stability of the protein-ligand complex using RMSD, RMSF and protein-ligand interactions. Therefore, it is of interest to report the molecular docking analysis of breast cancer target RAC1B with ligands. Here, data shows that the therapeutic compounds that were evaluated showed greater stability in comparison to the reported compounds, EHop-016 and has found promising medication possibilities for breast cancer that target Rac1b.

## Background:

One of the most prevalent cancers in women worldwide is breast cancer. Due to a number of risk factors related to bio-molecular dynamics, breast carcinogenesis is not well understood [1]. In 2022, 2.3 million women were diagnosed with breast cancer, and 670,000 died worldwide [2]. Cells with defective division and mutation due to genetic damage are the source of cancer and one kind of hormonal cancer is breast cancer [3]. It is essential to understand the molecular route that initiates and switches to cell migration [4]. Breast tissues are glandular in nature and are extremely responsive to variations in the body's hormone levels [5]. With 0.3 million fatalities annually, cancer is the second most deadly disease in India and the most common malignancies found in Indian populations are those of the breast, colon, lungs, liver, rectum, and stomach [6]. Cancer can be caused by any physical or chemical source; such as hormones, ionizing radiation, ingested asbestos, tobacco smoke, and way of life [7]. The cells' ability to recognize the signals for cell proliferation has been compromised by their modification of the cell signaling system, which permits uncontrollably rapid cell growth [8]. Defects in the apoptotic and cell cycle regulatory pathways occur in cancer cells as a result of stress or damage to DNA [9]. These factors promoted the growth, proliferation, and metastasis of cancer cells to different body parts [10]. Analysing these compounds' potential as in vitro inhibitors of microtubules or proteins that cause cancer can be a time-consuming and costly procedure that involves the use of expensive chemicals, cancer cell lines, and animal models [11]. Therefore, it becomes advantageous to use *in silico* modelling before evaluating these medications experimentally as implementing this method can save a lot of money, time, and energy [12]. The *in silico* approach is a genuinely amazing tool that lets us estimate possible drug candidates and their affinity for particular target regions while also estimating their metabolism and least amount of side effects [13]. Even though several proteins are connected to breast cancer, but I chose to concentrate on RAC1B since it is connected to a multitude of other cancers [14]. Also the recent study published in Nature revealed that Rac1 could be a priority target for cancer therapy, with statistics to support its feasibility [15].

The spread of breast cancers, tumor recurrence, and treatment resistance are thought to be caused by breast cancer stem cells (BCSC) [16]. However, the absence of BCSC-selective molecular targets and their heterogeneity have impeded the development of BCSC-targeting therapeutics [17]. Following a review of the literature, we discovered that RAC1B, the sole alternatively spliced version of the small GTPase RAC1, is expressed in vivo in a fraction of BCSCs and is necessary for the upkeep of BCSCs and plays a crucial role as a signalling node downstream of several micro-environmental signalling pathways, such as those that are started by growth factors and cell adhesion [18]. Rho GTPases regulate a variety of important biological activities, including actin dynamics, gene transcription, and cell cycle progression [19]. RAC1 signaling controls biological functions such tumor cell survival, proliferation, and invasion and is increased in a number of malignancies, including breast cancer [20]. Significantly, RAC1 is linked to tumor cells' resistance to target and cytoablative therapies [21]. However, there is minimal therapeutic significance for possible RAC1-targeted therapy due to its nearly universal expression and vital roles in numerous organ systems [22]. Hyperactivation of RAC1 signaling in cancers is caused by rare mutations, overexpression, or dysregulation by RAC1-regulatory proteins, such as GTPase-activating proteins (GAPs), guanine-nucleotide exchange factors (GEFs), and guanine-nucleotide dissociation inhibitors (GDIs) [23]. The alternate splicing of RAC1 to produce the constitutively active RAC1B variant, a small GTPase, contributes to the hyperactivation of RAC1 signaling in certain solid tumors [24]. The structures of Rac1b in the GDP- and GppNHp-bound forms demonstrate that the insertion leads to a highly mobile switch II and an open switch I conformation at a resolution of 1.75 A [25]. An extra exon, exon3b, has been found in RAC1B [26]. It encodes 19 amino acids and features an in-frame insertion right after the Switch-II domain [27]. As a result, a conformational shift that is independent of GEF-mediated activation and favors the active GTP-bound state occurs [28]. For BCSCs, a subset of cancer cells with stem-like characteristics, to survive and function, RAC1B is essential [29]. Tumor start, metastasis (spread), and post-treatment recurrence are believed to be caused by these BCSCs [30]. Research has indicated that BCSCs lack the ability to withstand chemotherapy and grow tumors when they lack RAC1B [31]. Chemotherapy medications such as doxorubicin are less effective against breast cancer cells due to the presence of RAC1B [32]. Chemotherapy can more effectively treat breast cancer cells when RAC1B is not present [33]. In general, RAC1B is becoming a significant target for the creation of novel treatments for breast cancer [34]. By blocking RAC1B, scientists intend to target BCSCs and enhance the efficacy of chemotherapy, which could result in better treatment outcomes [35]. The structure-based drug design approach has been used in this study. The whole set of experimental drugs with strong binding affinities was screened from DrugBank. In addition, A selected group of molecules undergo Molecular Docking studies to identify the specific type of protein-ligand interaction. Next, in order to find strong inhibitors of RAC1B linked to breast cancer, a group of medications is subjected to property analysis of Absorption, Distribution, Metabolism, and Excretion (ADME) and Molecular Dynamics Simulations (MDS) in comparing with the reported compound EHop-016.

## Material and methods:

## Protein preparation:

The Protein Data Bank (PDB) website contains a database of three-dimensional structures of large biological molecules, including proteins and nucleic acids. The structure of the RAC1B protein was retrieved from the protein data bank (https://www.rcsb.org/pdb) at a resolution of 1.75 A° and PDB ID 1RYF [36]. Two identical protein chains, A and B, make up the arrangement 1RYF [37]. The PDB files that were first downloaded do not have the proper bonding configuration and do not contain adequate hydrogen atoms to be useful for any future studies. Thus, the "MG Tools of AutoDockVina program and Biovia Discovery Studio software" were used to resolve all concerns and prepare the proteins and generate a ready-to-dock protein [38]. With Autodock Vina, all interacting heavy atoms, water molecules, and metal ions are eliminated and replaced with hydrogen atoms. Charges for Kollman were assigned. The final macromolecule structure was modified by adding solution parameters using AutoDock's Addsol function [39]. The protein's structure was saved in PDB format for future studies.

## Ligand preparation:

By identifying different ligands, one can understand the activity of a receptor or target protein. In this case, the whole experimental drug library was screened, and out of many, thirty experimental chemical compounds were collected in PDB format from the DrugBank database for further study. DrugBank (https://go.drugbank.com/) is an essential tool for any pharmaceutical research since it offers reliable and accurate medication data that is arranged for easy program integration or fast access [40]. The ligand was synthesized by a series of steps including 2D-3D conversions, structural correction, the synthesis of variants of these structures, and the optimization and verification of the structure [41]. After being downloaded in PDB format, the top 30 drug structures were transformed to a PDBQT file format so that the AutoDock software could access and recognize them. Using AutoDock Tools 4.2.6 (https://ccsb.scripps.edu/mgltools/), each ligand selected from the simulated screening procedure is prepared [42]. Every ligand was made using the steps mentioned as follows: After the addition of Gasteiger charges, the integration of non-polar hydrogen bonds, the identification of rotatable bonds and aromatic carbons, and the activation of TORSDOF [43]. Now the file is being saved in PDBQT format. Furthermore, docking studies have been performed with these compounds.

## Active site prediction:

One of the most important tasks in the drug development process is predicting the functional active site from the protein's tertiary structure [44]. The site map module of the Schrodinger program (https://www.schrodinger.com/platform/products/sitemap/) has been used to identify the protein's active site [45]. SiteMaps aid in the identification and druggability assessment of binding sites, including protein-protein interfaces and allosteric binding sites [46]. Apart from its influence on lead generation, SiteMap can help investigators optimize leads by offering perceptions into potential ligand-receptor relationships, which can subsequently direct the alteration of lead compounds to boost their binding ability [47]. In this case, all possible sites for the sections of the target protein were grouped according to the site score.

## Molecular docking:

When figuring out the structure and evaluation of a small-molecule ligand-protein interaction in a complex, molecular docking is an effective technique [48]. It is applied to investigate the behavior of molecules when target proteins bind. Also, it offers a wide set of sample options and is a rapid and easy technique to screen huge collections of ligands and targets [49]. It is a technology that is widely used in the search for new drugs. Top docking software includes AutoDock, Vina, PyRx MOE-Dock, FLexX, and GOLD [50]. In this study, AutoDock Vina has been used to perform molecular docking. Using AutoDock Vina (https://vina.scripps.edu/), molecular docking has been carried out in this work wherein the ligands were docked to the protein active sites to assess the selected compounds' affinity for atomic binding [51]. The initial grid parameters were as follows: Size Z = 20, Size X = -2.444440, Center Z = 35.521089, Center Y = 69.964664. The active site-specific docking is aided by these parameters. The docking simulations employed an exhaustiveness parameter of 8, an energy range, and a random seed. The docked complexes were loaded into BIOVIA Discovery Studio Visualizer for further analysis and display of the active amino acid residues and 2D-3D interaction diagram [52].

## ADME Properties:

The main task in drug design is to identify one or more molecules that have the desired effects on medicines. The chemical must not only have a high affinity for the target protein but also be able to reach the site of action, exhibit acceptable drug-like characteristics, and have a suitable selectivity profile [53]. Absorption, Distribution, Metabolism, Excretion, and Toxicity are all referred to as ADMET. It determines a compound's (drug molecule's) pharmacodynamic actions and contains the compound's pharmacokinetic profile [54]. The qualities of the active compounds, including their oral absorption, brain penetration, bioavailability, and other human intestinal absorption properties, have been assessed using SwissADME (http://www.swissadme.ch/) and pkCSM (https://biosig.lab.uq.edu.au/pkcsm/), taking into consideration all three of the active screened compounds and the reported compound [55]. The physicochemical parameters and ADME-T profile of the top thirty compounds were predicted using their canonical SMILES. The compounds' SMILES format was taken from the DrugBank database (https://go.drugbank.com/). The drug-likeness was also evaluated using QikProp-V6, Glide v8.3, and Schrodinger, LLC, New York, NY, 2020-4 [56].

## Molecular Dynamics Simulation:

Molecular dynamics (MD) simulations are becoming more and more helpful in modern drug development process [57]. The main aim of MD simulations is to ascertain the stability of chemical compounds during the development of drug-protein complexes [58]. Using the Desmond package and the force field OPLS2005 (Schrödinger, LLC, New York, NY, 2015), the protein structure of RAC1B (PDB code: 1RYF) and the docked complexes of the top three screened ligands and reported ligand were simulated using MD force field over a 100 ns time frame. Na + and Cl-ions were introduced to the neutralization system, and the TIP3P water cube model was utilized for solvation [59]. To avoid collision with its periodic image, the protein complex is positioned 10 Å away from the box wall. Using the steepest descent algorithm, energy minimization was accomplished for a maximum of 50,000 steps [60]. The structures stabilized at a maximum force of 1000 kJ mol-1 nm-1 after two-step equilibrium at 300 K, 1.0 atm air pressure, and 50,000 steps [61]. The final production process was kept running at 300 K, with pressure held at 1.01325 atm, and time steps of 100 ns [62]. Following the simulation, the root mean square deviation (RMSD) and root mean square fluctuation (RMSF) will be evaluated using the visual molecular dynamics (VMD) software [63].

## Results and discussion:

## Active site prediction:

**Figure 1:**
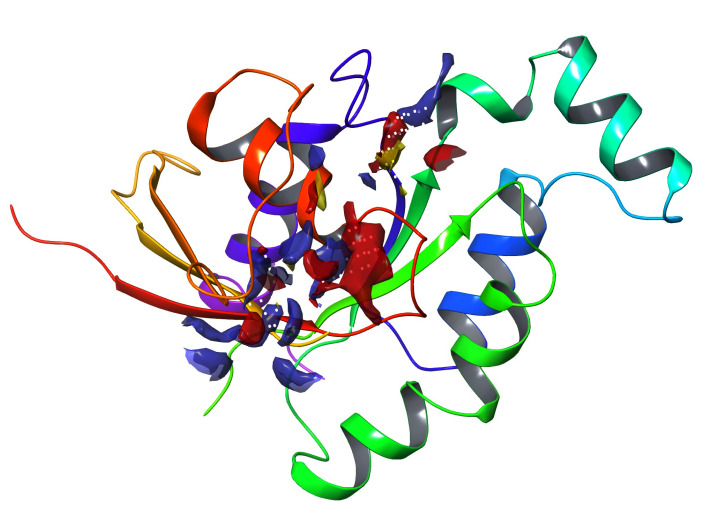
Active site of 1ryf

Based on the site score, high-scoring clusters were selected from an active site that was projected using the sitemap. The druggability value (D-Score) for RAC1B is 0.824, and its site score is 0.883 and the binding site's residues are ASP11, GLY12, ALA13, VAL14, GLY15, LYS16, THR17, CYS18, LEU19, PHE28, TYR32, ILE33, PRO34, THR35, PHE37, ASP38, TYR40, ASP57, THR58, ALA59, THR134, LYS135, ASP137, LEU138, CYS176, SER177, ALA178, LEU179. Table 1 displays the calculated binding site properties, and Figure 1 shows the projected binding site residues.

## Molecular Docking:

**Figure 2:**
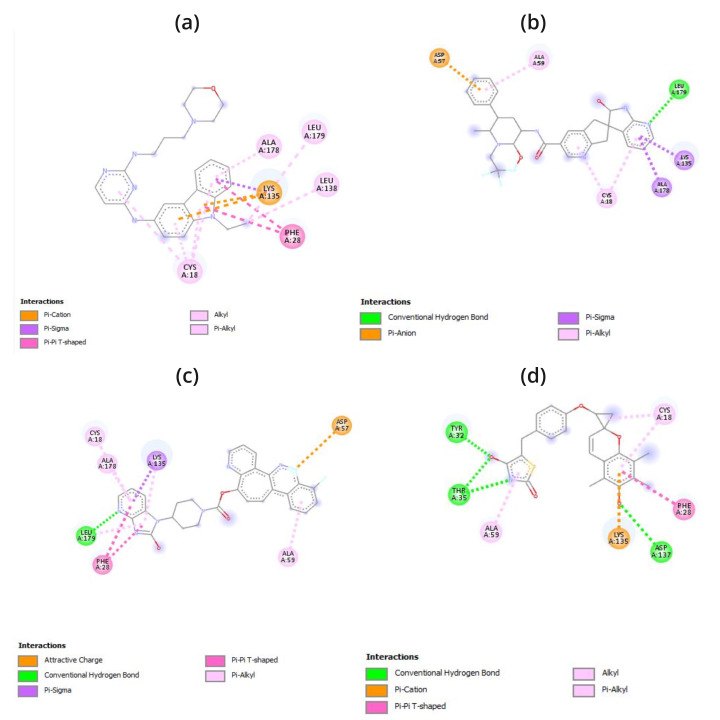
2D interaction diagram of 1ryf with (a) EHop-016, (b) DB15328, (c) DB12457, (d) DB00197

**Figure 3:**
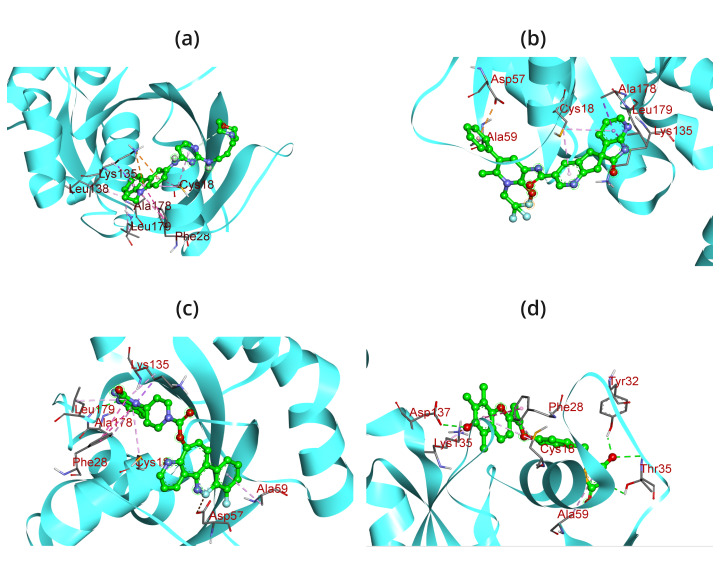
3D interaction diagram of 1ryf with (a) EHop-016, (b) DB15328, (c) DB12457, (d) DB00197

The atomic-level interaction between a small molecule and a protein has been replicated by researchers using the molecular docking technique. This has allowed them to determine the binding energy of small molecules in the binding region of target proteins and has provided insight into the underlying efficiency required [64]. The binding energy of the peptide was determined by performing docking computations utilizing the AutoDock (PyRx). The docking scores are recorded in Table 2 which range from -11.6 to -9.7 in case of screened compound and -8 of reported compound EHop-016. It was discovered that each of these substances binds to ATP-binding residues located in the target protein's active site. It indicated the drug molecules' stronger binding to the target proteins' active regions and their enhanced inhibitory effects [65]. The newly found molecules were shown to be involved in both hydrophobic and hydrogen bond interaction residues; finally, the most effective compounds were identified by comparing their binding energies. As a result, the hydrogen bond interaction demonstrates the ligand's high selectivity for the target and its good affinity for binding the target protein that contains residues of the necessary amino acids. As a result, the hydrogen bond interaction demonstrates the ligand's high selectivity for the target and its good affinity for binding target proteins that contain residues of the necessary amino acids. The BIOVIA Discovery Studio application program has been used to create the interaction diagrams for hydrogen bonding, drug-protein interactions, and molecular docking pockets [66]. The results of this study show that docking 1RYF with the top three compounds produced the following results, conventional hydrogen bonds (LEU A:179, TYR A:32, THR A:35, ASP A:137), Pi-Anion (ASP A:57), Pi-Sigma (ALA A:178, LYS A:135), Pi-Alkyl (ALA A:59, CYS A:18, ALA A:178), attractive charges (ASP A:57), Pi-Pi T-shaped (PHE A:28), and Pi-Cation (LYS A:135) and reported compound make ALA178, LEU138, LEU179, LYS135, CYS18, PHE28 respectively which is given in the Table 2, Figure 2 & Figure 3.

## ADME Properties:

The 30 selected experimental drug compounds were obtained from DrugBank, and their physiochemical properties and drug-likeness were determined employing pkCSM (https://biosig.lab.uq.edu.au/pkcsm/) and the Swiss ADME web server (Swiss Institute of Bioinformatics, Switzerland) given in the (Table 3). The drugs' toxicity, excretion, metabolism, distribution and absorption were also examined using QikProp (ADMET). Qikprop was used on a group of recently produced compounds. The identified compounds under research had ADME properties that made them excellent choices for drug development according to the Qikprop module [67]. The drug-likeness was evaluated using QikProp-V6, Glide v8.3, and Schrodinger, LLC, New York, NY, 2020-4 with the help of the following attributes: molecular weight of the compound (130.0-725 Da), the number of hydrogen bond acceptors (2.0-20.0), the number of donor hydrogen bonds (0.0-6.0), and the number of rotatable bonds (0.0-15.0), Area of surface (7-200), QP log BB Permeability (-3.0 to 1.2), QP log Po/w (<5), QPPCaco (>0.9) and CNS Permeability (-2 to +2%), Human oral absorption as a percentage (>80% is high, <25% is poor) [68]. It is thought that each of these traits, along with molecular flexibility, significantly influences oral bioavailability. Thus, less frequently, the acquired ADMET properties of both screened and reported compounds are fall within the suggested ranges. Based on the QikProp module, three drug compounds (DB15328, DB12457, and DB00197) out of 30 were chosen for the next step, and the compounds that do not meet the ADME parameters are eliminated from the study.

MW - Molecular weight of the molecule = 130.0-725 Da.

DHB - Number of donor hydrogen bonds = 0.0-6.0.

AHB - Number of hydrogen bond acceptors = 2.0-20.0.

RB - No. of Rotatable Bonds = 0.0-15.0

PSA - Surface area of polar nitrogen and oxygen atoms and carbonyl carbon atoms = 7-200

QP log Po/w - Predicted octanol/water partition coefficient = <5

QPPCaco - Predicted apparent Caco-2 cell permeability in nm/sec. = >0.9

QP log BB Permeability - Predicted brain/blood partition coefficient = -3.0 to 1.2

CNS Permeability = -2 to +2

% HOA - Percentage of human oral absorption = >80% is high, <25% is low

## Molecular Dynamics Simulation:

**Figure 4:**
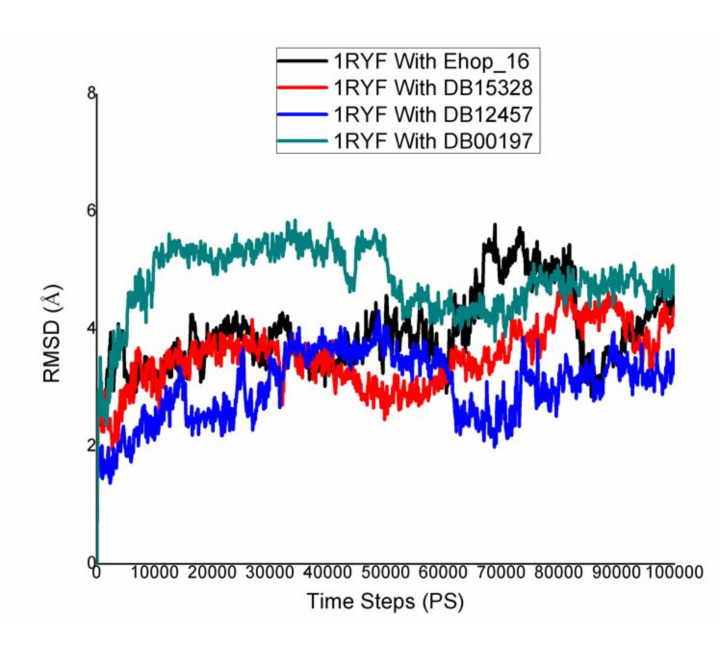
RMSD graph of 1ryf with reported (EHop-016) and screened compounds (DB15328, DB12457, DB00197)

**Figure 5:**
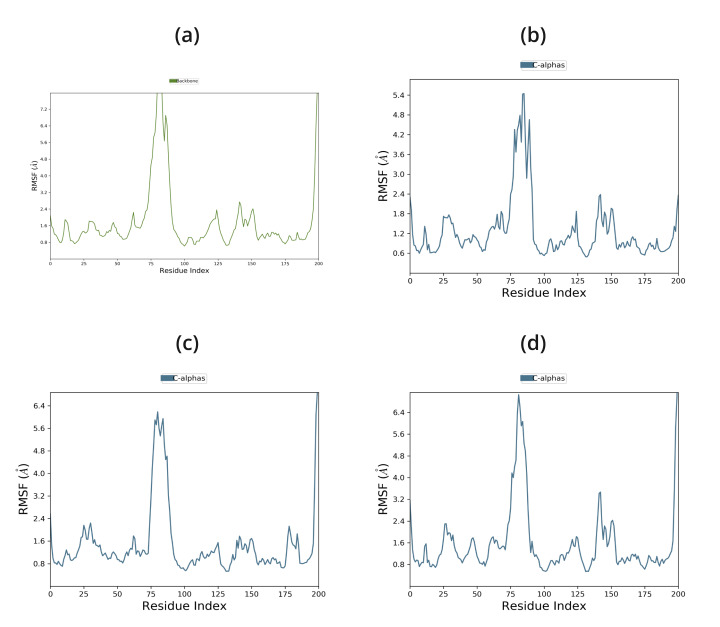
RMSF Graph of 1ryf with (a) EHop-016, (b) DB15328, (c) DB12457, (d) DB00197

**Figure 6:**
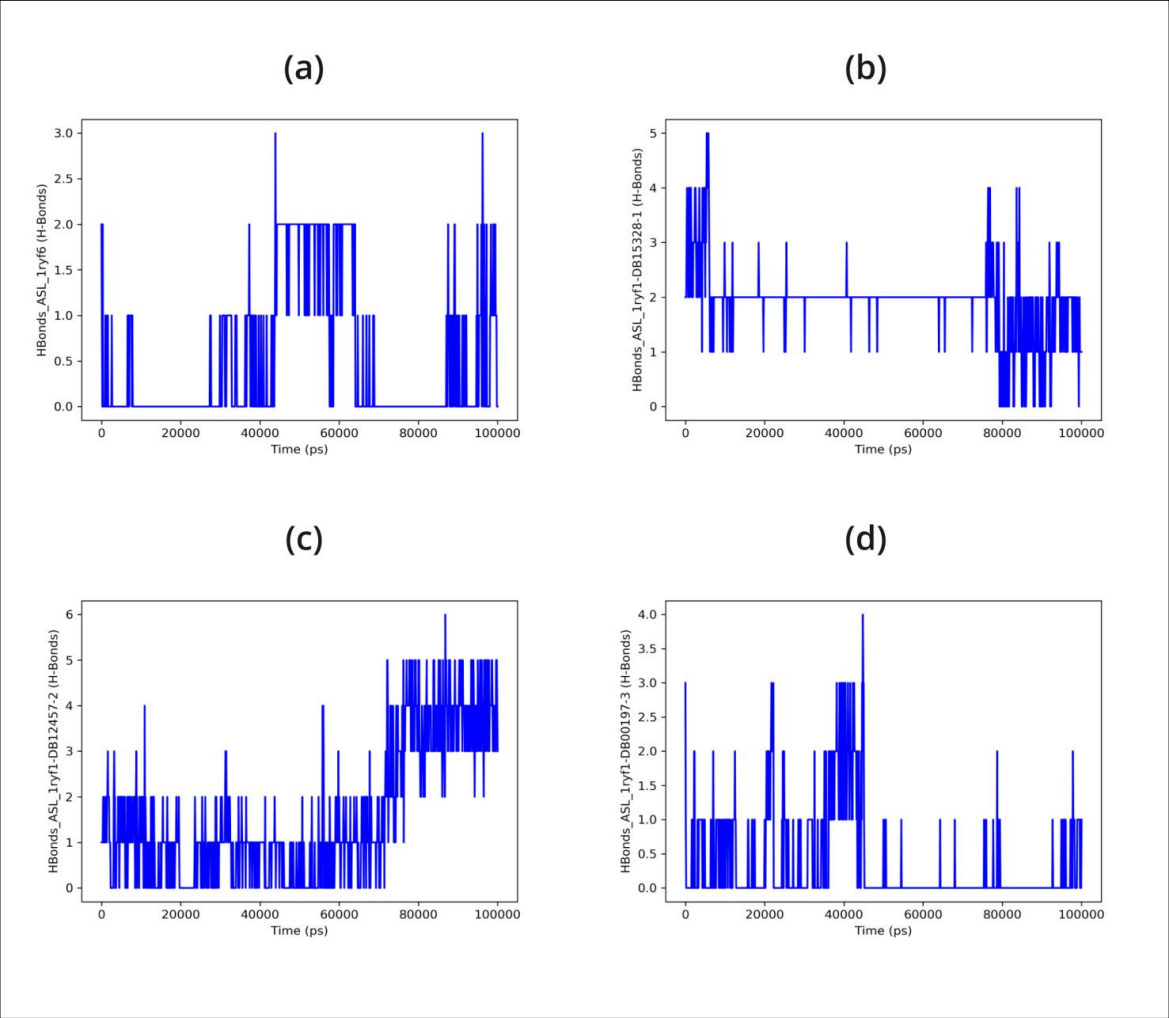
Hydrogen bond interaction of 1ryf with (a) EHop-016, (b) DB15328, (c) DB12457, (d) DB00197

**Figure 7:**
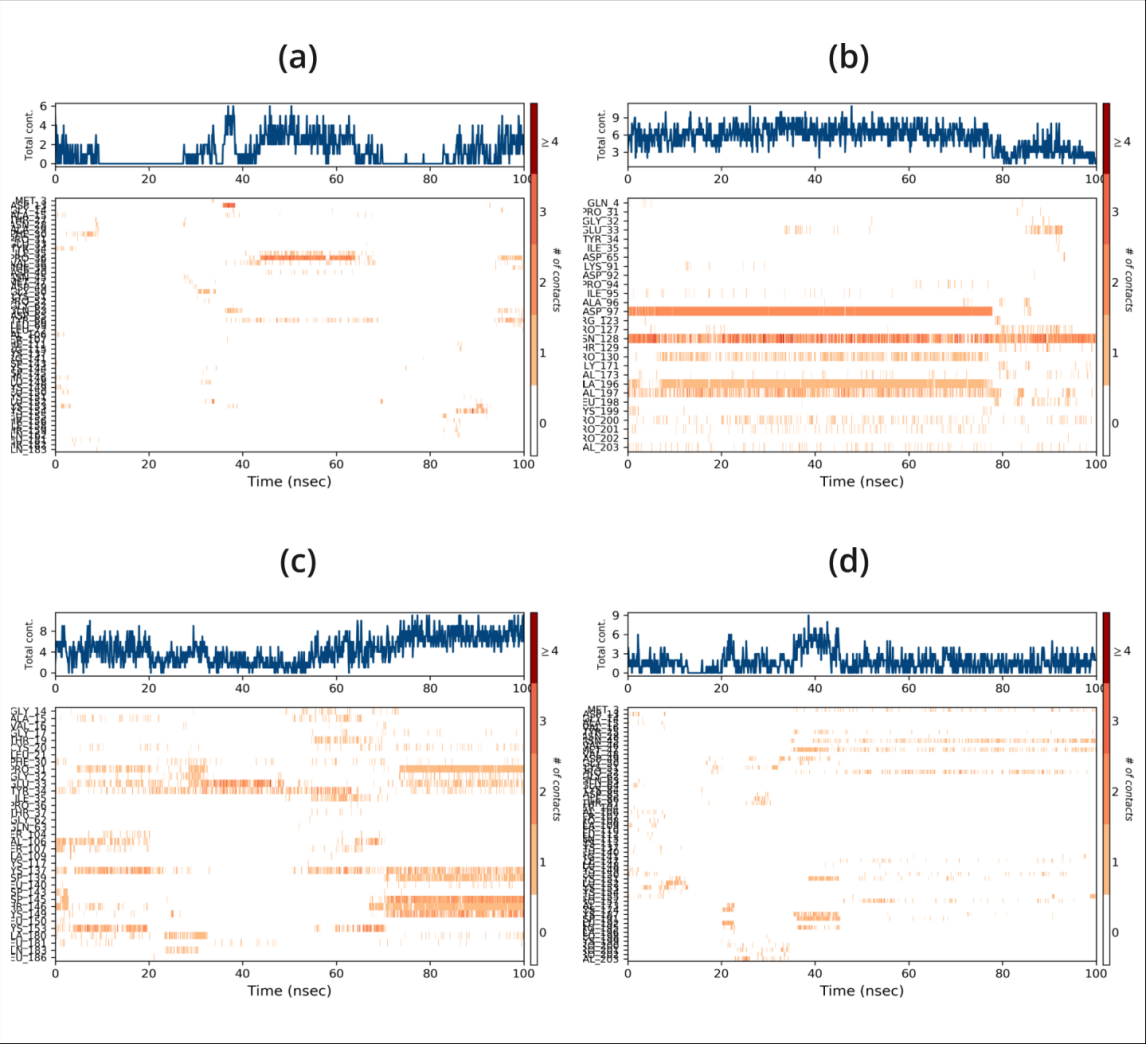
Protein-ligand timeline of 1ryf with (a) EHop-016, (b) DB15328, (c) DB12457, (d) DB00197

**Figure 8:**
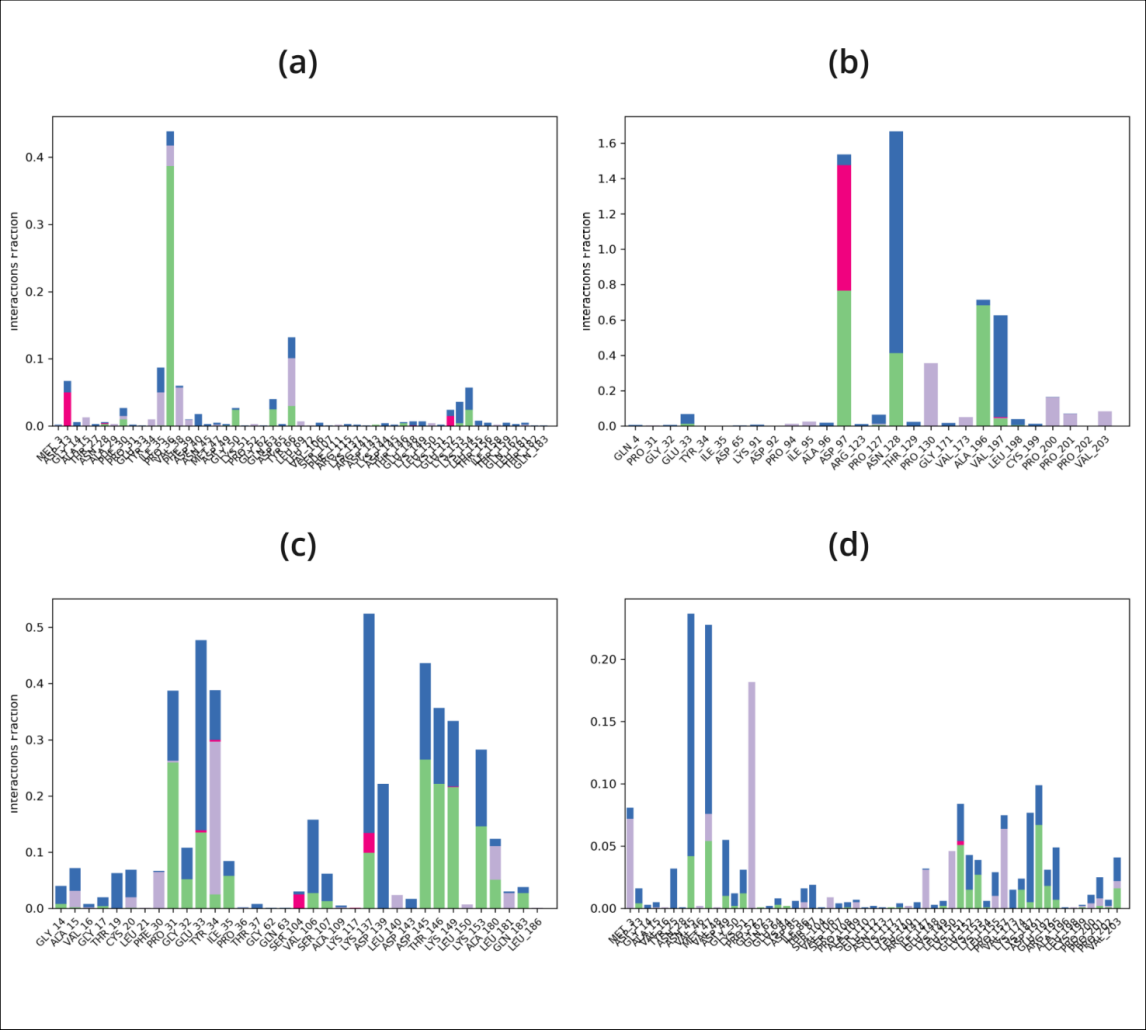
Protein-Ligand Histogram of 1ryf with (a) EHop-016, (b) DB15328, (c) DB12457, (d) DB00197

Protein-ligand complexes were simulated in order to demonstrate high stability at the compound's simulation point and to ascertain the accuracy of the docking process in terms of average RMSD and RMSF. The binding pose in the corresponding crystal structures, ligand, and protein interaction complex is represented by this value. Desmond was utilized to examine the simulation of molecular dynamics. The Desmond suite in Schrodinger has been utilized for performing molecular dynamics simulations for 100 ns in order to obtain information about the stability of the protein-ligand pair that is best-docked. The screened compounds DB15328, DB12457, and DB00197 fluctuate at starting and continue to do so till 60 ns, after which all of the protein-ligand complexes become stable over the course of the simulation, as shown by the Root Mean Square Deviation (RMSD) of the protein-ligand complex; however, the reported compound EHop_016 fluctuated throughout the simulation time shown in Figure 4. All of the screened compounds' active site residues showed less variation in the Root Mean Square Fluctuation (RMSF) data than the reported compound, which fluctuated greatly between 60 and 100 ns, as shown in Figure 5. Furthermore, all of the screened compounds, particularly (b) and (c), maintain the hydrogen bond interaction consistently, even with the fact that there was a loss of hydrogen bond interaction between the ligand and the protein target during the simulation time. At last, however, all of the compounds are interacting with the protein target during the 100 ns simulation time, as shown in Figure 6 & Figure 7. Finally, the hydrogen bond and hydrophobic bond residue contribution for all the screened compounds shows better stability as compared with the reported compound, as given in Figure 8. Overall, it is evident from the molecular dynamics simulation results that the screened compounds shows superior protein-ligand binding stability compared to the reported compound EHop_016 in all respects.

## Conclusion:

With the availability of high-performance computers that enable the processing of larger and more complicated data sets, in-silico methodologies for drug creation have undergone a revolutionary change due to recent advancements in computational software and hardware. Here, we employed a structure-based drug design strategy to identify the most effective lead compounds against Rac1b in breast cancer. Based on the outcomes of molecular docking, drug likeness analysis, metabolism, excretion, absorption, distribution, and toxicity (ADMET) assessment, and molecular dynamics simulation with the three screened and one reported compound, EHop-016, it is possible to determine that the three drug compounds (DB15328, DB12457, and DB00197) can be noted as powerful lead molecules that have a high binding affinity and specificity, enabling them to bind to the target strongly, which could be a better inhibitor for Rac1b in breast cancer in the future.

## List of Abbreviations:

PDB = Protein Data Bank

SBDD = Structure-Based Drug Design

ADME = Absorption, Distribution, Metabolism, Excretion

MDS = Molecular Dynamics Simulation

RMSD = Root Mean Square Deviation RMSF = Root Mean Square Fluctuation HBA = Hydrogen Bond Acceptor HBD = Hydrogen Bond Donor

HOA = Percentage of human oral absorption

PSA = Polar Surface Area

RB = Rotatable Bonds

MW = Molecular Weight

DHB = Donor Hydrogen Bonds

AHB = Acceptors Hydrogen Bonds

BB = Brain/Blood

CNS = Central Nervous System

PL = Protein Ligand

HB = Hydrogen Bond

VMD = Visual Molecular Dynamics

## Figures and Tables

**Table 1:** Active site prediction parameters of 1ryf

**Site Score**	**D Score**	**Volume**	**Size**	**Residue**
0.883	0.824	208.201	82	ASP11, GLY12, ALA13, VAL14, GLY15, LYS16, THR17, CYS18, LEU19, PHE28, TYR32, ILE33, PRO34, THR35, PHE37, ASP38, TYR40, ASP57, THR58, ALA59, THR134, LYS135, ASP137, LEU138, CYS176, SER177, ALA178, LEU179

**Table 2:** Results of molecular docking of 1ryf with screened compounds

**S.No**	**Compounds**	**Affinity Docking Score**	**Hydrogen Bond Interaction**	**Hydrophobic**
Reported Compound:
1	EHop-016	-8	----	ALA178, LEU138, LEU179, LYS135, CYS18, PHE28
**Screened Compounds:**
1	DB01126	-11.6	ILE A:33, LYS A:135, LEU A:19, THR A:134, GLY A:15, ALA A:178	PHE A:28, CYS A:18,
2	DB09280	-11.2	ALA A:178, LYS A:135, GLY A:15, ALA A:13, VAL A:14, LYS A:16, THR A:134	CYS A:18, LEU A:179, PHE A:28
3	DB09374	-11.1	LYS A:135, ALA A:178, LEU A:179	TYR A:32, ALA A:13, CYS A:18
4	DB15328	-10.9	LEU A:179	ASP A:57, ALA A:59, LYS A:135, ALA A:178, CYS A:18
5	DB11611	-10.7	GLY A:15, THR A:35, TYR A:32, ASP A:137	LEU A:138, LEU A:179, LYS A: 135, PHE A:28, ALA A:59
6	DB00872	-10.5	--	ALA A:178, PHE A:28, LYS A:135, CYS A:18, LEU A:179, ALA A:59
7	DB04868	-10.5	ILE A:33, LYS A:16, ALA A:13, TYR A:32, CYS A:18, GLU A:31	LEU A:179, LEU A:138, LYS A:135
8	DB09297	-10.4	GLY A:30, PRO A:29, LYS A:135, SER A:102, SER A:105	ALA A:13
9	DB01396	-10.3	PHE A:97, ASP A:57, THR A:35, TYR A:32, THR A:134, LYS A:135, LEU A:179	ALA A:178, ALA A:59
10	DB09143	-10.3	LEU A:138, LEU A:179, ALA A:178, CYS A:18, ASP A:57, PHE A:37, PRO A:34, THR A:35, ASP A:38	LYS A:135, ALA A:59
11	DB00320	-10.3	PHE A:28, ASP A:137, CYS A:18, ALA A:178, LYS A:135, GLY A:15, THR A:17	LEU A:138, LEU A:179
12	DB12457	-10.2	LEU A:179, ASP A:57	CYS A:18, ALA A:178, LYS A:135, PHE A:28, ALA A:59
13	DB08901	-10.1	ILE A:33, CYS A:18, GLY A:15	PHE A:28, LYS A:135, TYR A:32, ALA A:59
14	DB09074	-10.1	ILE A:33, TYR A:32, ALA A:13, GLY A:15, ALA A:178, LEU A:179, ALA A:59	CYS A:18, PHE A:28, LYS A:135, LEU A:138
15	DB06210	-10.1	ALA A:13, LYS A:16, THR A:17, GLY A:12, LEU A:138, LEU A:179	LYS A:135, ALA A:178, PHE A:28, CYS A:18,
16	DB01251	-10.1	THR A:17, GLY A:15, ILE A:33	PHE A:28, LYS A:135, LEU A:179, CYS A:18, ALA A:59
17	DB13345	-10.1	THR A:17, GLY A:15, ILE A:33	PHE A:28, LYS A:135, LEU A:179, CYS A:18, ALA A:59
18	DB15233	-10.1	TYR A:32, THR A:17, ASP A:137, PHE A:37, ASP A:57	CYS A:18, ALA A:59
19	DB01419	-10	THR A:35, ASP A:57, PHE A:37, PRO A:34, ASP A:137, LEU A:138, LEU A:179	LYS A:135, ALA A:178, PHE A:28, CYS A:18, ALA A:59, ILE A:33
20	DB13954	-9.9	ILE A:33	PHE A:28, LYS A:135, ALA A:178, CYS A:18
21	DB00696	-9.9	GLY A:15, THR A:17, ASP A:137, PHE A:28, ALA A:178, LYS A:135, CYS A:18	TYR A:32, LEU A:179, LEU A:138
22	DB08827	-9.9	TYR A:32, LYS A:135, LEU A:19, GLY A:15, THR A:134	LEU A:179, PHE A:28, ALA A:178, CYS A:18
23	DB00984	-9.8	--	LYS A:135, ALA A:178, PHE A:28, CYS A:18
24	DB01259	-9.8	LYS A:135, GLY A:15	ALA A:178, PHE A:28, CYS A:18, ALA A:13, TYR A:32
25	DB13520	-9.8	ILE A:33, GLY A:15, LYS A:135	CYS A:18, PHE A:28, ALA
26	DB11274	-9.7	--	PHE A:28, LEU A:179, LYS
27	DB11652	-9.7	CYS A:18, GLY A:15, TYR A:32, ALA A:178, LEU A:179, LYS A:135,	PHE A:28, ILE A:33, ALA A:59
28	DB00197	-9.7	TYR A:32, THR A:35, ASP A:137	ALA:59, CYS A:18, PHE A:28, LYS A:135
29	DB08995	-9.7	GLY A:15, LYS A:16, THR A:17, THR A:35, PRO A:34, LEU A:179	ALA A:13, ALA A:59, PHE A:28, LYS A:135, CYS A:18
30	DB00471	-9.7	LYS A:16, ALA A:13, VAL A:14, GLY A:15	PHE A:28, ALA A:178, CYS A:18, LYS A:135, TYR A:32, ALA A:59

**Table 3:** ADMET Properties of reported and screened compounds

**S. No.**	**Compound ID**	**Mol. Weight (130-725)**	**LogP (<5)**	**Rotatable Bonds (0-15)**	**Acceptors (2-20)**	**Donors (0-6)**	**Surface Area (7-200)**	**Caco2 permeability (>0.9)**	**Intestinal absorption >80% high/ <25poor**	**BBB permeability (-3.0-1.0)**	**CNS permeability (-2 to +2)**
**Reported Compound:**
1	EHop-016	430.5	4.48	8	7	2	187.9	0.834	91.52	0.201	-2.337
**Screened Compounds:**
1	DB15328	549.5	3.53	4	5	2	227.5	0.923	98.29	-1.039	-2.53
2	DB12457	534.5	4.49	3	7	2	222.1	1.954	92.47	-1.747	-3.271
3	DB00197	441.5	4.37	5	6	2	185.8	0.776	92.07	-0.586	-2.15

## References

[1] Chen W (2016). CA Cancer J Clin..

[2] https://www.who.int/news-room/fact-sheets/detail/breast-cancer.

[3] Davis JD, Lin SY (2011). World J Clin Oncol..

[4] Singh AK (2024). Sci Rep..

[5] Akram M (2017). Biological Research..

[6] Yadav M (2020). Current Drug Discovery Technologies..

[7] Rajabi S (2021). Biomolecules..

[8] Mathews FS (1933). Ann Surg..

[9] Hulka BS (1996). Prog Clin Biol Res..

[10] Rahman MM (2022). Biomedicine & Pharmacotherapy,.

[11] Bellanger M (2018). J. Glob. Oncol..

[12] Kazmi SR (2019). Comput. Biol. Med..

[13] Nagini S (2017). Anticancer Agents Med Chem..

[14] Hall A, Nobes CD (2000). Phil. Trans. R. Soc. Lond. B Biol. Sci..

[15] Li X (2020). Front Pharmacol..

[16] Colditz GA (2012). Breast Cancer Res Treat..

[17] Chen F (2023). Oncogene..

[18] Fritz G (2002). Br J Cancer..

[19] Cardama GA (2014). Anti-cancer Agents in Medicinal Chemistry..

[20] Kamai T (2004). Clin Cancer Res..

[21] De P (2019). Cells..

[22] Olabi S (2018). Breast Cancer Res..

[23] Marei H, Malliri A (2017). Small GTPases..

[24] Schnelzer A (2000). Oncogene..

[25] Gamblin SJ, Smerdon SJ (1998). Curr Opin Struct Biol..

[26] Bagci H (2014). Cell Death Dis..

[27] Jordan P (1999). Oncogene..

[28] Hein AL (2016). Oncogene..

[29] Singh A (2004). Oncogene..

[30] Ungefroren H (2014). Oncotarget..

[31] Rosenblatt AE (2011). Endocr Relat Cancer..

[32] Tsou S-H (2015). PLoS One..

[33] Zhou C (2013). Oncogene..

[34] Melzer C (2017). Cell. Commun. Signal..

[35] Wang X (2018). Scientific reports.

[36] Sung H (2021). CA Cancer J Clin..

[37] Cardama GA (2018). Crit Rev Oncol Hematol..

[38] Pushpalatha R (2017). Journal of Young Pharmacists..

[39] Pushpalatha R (2017). Journal of Drug Delivery Science and Technology..

[40] Lai H-W (2012). Evidence-Based Complementary and Alternative Medicine..

[41] Palma G (2015). Oncotarget..

[42] Motta S, Bonati L (2017). J. Chem. Inf. Model..

[43] Ropp PJ (2019). J. Cheminformatics.

[44] Singh T (2011). J Chem Inf Model..

[45] Sankar K (2022). Mol Inform..

[46] Tavella D (2022). PLoS One..

[47] Beard H (2013). PLoS ONE..

[48] Tabassum S (2014). European journal of medicinal chemistry..

[49] Yun CH (2007). Cancer Cell..

[50] Singh AN (2017). Scientific reports.

[51] Dash R (2015). Bioinformation..

[52] Englebienne P (2007). Proteins..

[53] Matondo A (2022). Adv Appl Bioinform Chem..

[54] Cheng F (2012). J. Chem. Inf. Model..

[55] Daneman R, Prat A (2015). Cold Spring Harb Perspect Biol..

[56] Elmeliegy M (2020). Clin Pharmacokinet..

[57] Pang YT (2017). Journal of chemical theory and computation..

[58] Kawsar S (2022). Organic Communications..

[59] James PC (2020). Journal of Chemical Physics..

[60] Skjevik A (2015). Chemical Communications..

[61] Sehgal SA (2018). Tropical Journal of Pharmaceutical Research..

[62] Humphrey W (1996). Journal of molecular graphics.

[63] Bera I, Payghan PV (2019). Curr Pharm Des..

[64] Ali A (2023). J Mol Model..

[65] Du X (2016). Int J Mol Sci..

[66] Rehman MU (2023). Biomol Struct Dyn..

[67] Noor E-HD (2021). Current Drug Metabolism..

[68] Bai Z, Gust R (2009). Arch Pharm..

